# Different viral genes modulate virulence in model mammal hosts and *Culex pipiens* vector competence in Mediterranean basin lineage 1 West Nile virus strains

**DOI:** 10.3389/fmicb.2023.1324069

**Published:** 2024-01-17

**Authors:** Lise Fiacre, Antoine Nougairède, Camille Migné, Maëlle Bayet, Maxime Cochin, Marine Dumarest, Teheipuaura Helle, Antoni Exbrayat, Nonito Pagès, Damien Vitour, Jennifer P. Richardson, Anna-Bella Failloux, Marie Vazeille, Emmanuel Albina, Sylvie Lecollinet, Gaëlle Gonzalez

**Affiliations:** ^1^UMR VIRO, ANSES, ENVA, INRAE Virologie, Laboratoire de Santé Animale, Maisons-Alfort, France; ^2^UMR ASTRE, CIRAD, Petit-Bourg, Guadeloupe; ^3^Unité Des Virus Emergents (UVE), Aix-Marseille Université, IRD 190, INSERM 1207, Marseille, France; ^4^ASTRE, CIRAD, INRAe, Université de Montpellier, Montpellier, France; ^5^Institut Pasteur, Université Paris Cité, Arboviruses and Insects Vectors, Paris, France

**Keywords:** *Culex pipiens*, host virulence, Mediterranean basin, reverse genetics, vector competence, West Nile virus lineage 1

## Abstract

West Nile virus (WNV) is a single-stranded positive-sense RNA virus (+ssRNA) belonging to the genus *Orthoflavivirus*. Its enzootic cycle involves mosquito vectors, mainly *Culex*, and wild birds as reservoir hosts, while mammals, such as humans and equids, are incidental dead-end hosts. It was first discovered in 1934 in Uganda, and since 1999 has been responsible for frequent outbreaks in humans, horses and wild birds, mostly in America and in Europe. Virus spread, as well as outbreak severity, can be influenced by many ecological factors, such as reservoir host availability, biodiversity, movements and competence, mosquito abundance, distribution and vector competence, by environmental factors such as temperature, land use and precipitation, as well as by virus genetic factors influencing virulence or transmission. Former studies have investigated WNV factors of virulence, but few have compared viral genetic determinants of pathogenicity in different host species, and even fewer have considered the genetic drivers of virus invasiveness and excretion in *Culex* vector. In this study, we characterized WNV genetic factors implicated in the difference in virulence observed in two lineage 1 WNV strains from the Mediterranean Basin, the first isolated during a significant outbreak reported in Israel in 1998, and the second from a milder outbreak in Italy in 2008. We used an innovative and powerful reverse genetic tool, e.g., ISA (*infectious subgenomic amplicons*) to generate chimeras between Israel 1998 and Italy 2008 strains, focusing on non-structural (NS) proteins and the 3′UTR non-coding region. We analyzed the replication of these chimeras and their progenitors in mammals, in BALB/cByJ mice, and vector competence in *Culex (Cx.) pipiens* mosquitoes. Results obtained in BALB/cByJ mice suggest a role of the NS2B/NS3/NS4B/NS5 genomic region in viral attenuation in mammals, while NS4B/NS5/3′UTR regions are important in *Cx. pipiens* infection and possibly in vector competence.

## 1 Introduction

West Nile virus (WNV) is a mosquito-borne single-stranded, positive-sense RNA virus. It belongs to the family *Flaviviridae*, genus *Orthoflavivirus*. It is transmitted through an enzootic cycle involving birds as amplifying hosts and mosquitoes as vectors, with occasional spillovers to such mammals as humans and horses, these latter being considered to be dead-end hosts (Kramer et al., 2007). It is the most widely spread encephalitic flavivirus, having been reported in Africa, Europe, the Middle East, Western Asia (Li et al., 2013; Chowdhury and Khan, 2021) and America, as well as in Australia. It was first isolated from a native woman of Uganda in 1937 (Smithburn et al., 1940) and Africa is most presumably the cradle of WNV strains that are regularly introduced in Europe and the Middle East through bird migration. WNV was first detected in Israel in 1951 (Bernkopf et al., 1953), during which a high seroprevalence rate was demonstrated in the human population (Melnick et al., 1951). WNV circulation was also documented in Europe in France, Portugal and Cyprus in the 1960s (Lecollinet et al., 2019). Silent or paucisymptomatic infections lasted for 30 years before important human outbreaks and horse epizootics were first reported in Europe, Romania, Italy and Russia in 1996, 1998, and 1999, respectively. Since the 1990s, and even more frequently since 2010, WNV has actively circulated in the Mediterranean basin on many occasions (Murgue et al., 2001), such as in Northern Africa (Morocco, Tunisia, Algeria), in the Middle East (Israel, Turkey) and in Southern Europe (Italy, France, Spain, Croatia, Greece). A highly virulent lineage 1 WNV strain, classified among the Israelo-American clade, emerged in Israel in 1998, causing 35 human neuroinvasive cases in 1998 (Green et al., 2005), as well as the death of domestic geese and wild migratory birds, and in particular storks (Malkinson et al., 2002). Closely-related WNV lineage 1 isolates reemerged in Israel in 2018 and caused epizootics in birds and horses. While lineage 1 WNV belonging to the Western Mediterranean clade was evidenced as early as 1998 in Italy in Tuscany, regular WNV outbreaks in Italy associated to the circulation of such WNV strains had not been observed before 2008 in North-Eastern Italy (Emilia-Romagna, Veneto, and Lombardy), or before 2010 in South Italy. Of note, during the last decade, WNV lineage 1 strains have circulated sporadically in Italy, being apparently replaced by lineage 2 WNV; the new introduction of a closely-linked lineage 1 WNV strain coincided with an increased incidence of WNV neuroinvasive disease in humans in 2021/2022 in Northern Italy (Barzon et al., 2022). Finally, WNV has been (re)emerging throughout Europe for decades giving rise to unpredictable outbreaks of varying intensity in equine, human and avian populations with higher incidence of severe WNV cases in humans and horses, especially in 2018 (Martin and Simonin, 2019) and 2022 (Riccardo et al., 2022).

Bird migrations are mainly responsible for long-distance transmission and spread of the virus (Komar, 2003). Due to its ability to rapidly adapt to local environmental conditions, WNV has spread and emerged all around the world. Its circulation has been evidenced on all continents with the exception of Antarctica. Intense and recurrent WNV outbreaks of meningitis and encephalitis in birds, horses and humans have been reported in America, Africa or Europe. Although the majority of infected people remain asymptomatic (80%), a small proportion will develop flu-like symptoms (20%) or severe forms of the disease (1%), e.g., West-Nile Neuroinvasive Disease (WNND) characterized by meningitis, encephalitis or acute flaccid paralysis (Fiacre et al., 2020). WNV pathogenesis and WNV-induced lesions in humans and other susceptible mammals have been thoroughly characterized (Donadieu et al., 2013; Clé et al., 2020). Yet, our knowledge of the mechanisms underlying the ability of the virus to spread, to be transmitted with efficiency by European mosquitoes or to modulate pathogenicity in birds and mammals needs to be assessed in order to refine risk assessment and preparedness for epidemics in Europe (Fiacre et al., 2020).

West Nile virus Israel 1998 (WNV IS98) and WNV Italy 2008 (WNV IT08) are two Mediterranean lineage 1 strains that have caused outbreaks of varying intensity in the avifauna. While IS98 was highly virulent in domestic and wild birds, IT08 induced low mortality in the wild avifauna. This observation led us and others (Dridi et al., 2013) to suspect that genetic differences between the two strains may have accounted for differential virulence in birds. Dridi and colleagues performed experimental infections of specific-pathogen free (SPF) chicks and confirmed that the two strains differed in virulence in an avian model of WNV infection (Dridi et al., 2013), IS98 being more virulent than IT08. We provided the first description of the molecular determinants of WNV driving increased avian pathology of IS98 by using for the first time a classical reverse genetic approach and the generation of IS98-IT08 chimeras (Fiacre et al., 2023). Reverse genetics has been used for decades to identify amino acids involved in WNV virulence and has led to significant progress in understanding viral pathogenesis (Aubry et al., 2015). We initially used a plasmid method to create chimeras between the two parental strains IS98 and IT08 by replacing the NS4A, NS4B, NS5, and/or 3′ UTR regions of IS98 by the corresponding regions of IT08 and conducted *in vivo* experiments in mammals (BALB/cByJ mice) and birds (SPF chicks) (Fiacre et al., 2023). Genetic motifs implicated in differential pathogenesis in birds were consequently identified in the NS4A/NS4B/NS5 and 3′UTR regions. Our results suggest a role for the 3′ end of the WNV genome, especially the NS4A/NS4B/5′NS5 regions, in the decreased virulence of IT08 in SPF chickens, possibly due to the NS4B-E249D mutation. Other genetic markers could influence virulence of IS98 and IT08 in birds and mammals. Genome comparisons suggest a possible role of NS5-V258A, NS5-N280K, NS5-A372V, and NS5-R422K (not demonstrated) in attenuated phenotypes in mice.

A previous extensive review of the molecular determinants of WNV virulence in vertebrates and invertebrates indicated that critical motifs are disseminated all over the viral genome, not least in the 5′UTR and 3′UTR regions, and including key residues in the envelope (E) and non-structural 1 (NS1) proteins, E-159, NS1-175 or NS1-130 (Fiacre et al., 2020). Studies on molecular determinants of the virulence of European and Mediterranean WNV strains are scarce. In this context, the present study reports investigations on the role of structural and non-structural (NS) proteins, as well as non-coding genomic regions, in WNV transmission and virulence using the innovative reverse genetic ISA-method (*Infectious Subgenomic Method*) (Aubry et al., 2014). We designed six chimeric constructs between high- and low-virulence strains for birds, WNV IS98 and IT08, respectively, and tested them *in vitro* and *in vivo* on BALB/cByJ mice and on *Cx. pipiens*, the primary mosquito vector for WNV transmission in Europe and more specifically in the Mediterranean basin (Toma et al., 2008; Kampen et al., 2020; Tsioka et al., 2022).

## 2 Materials and methods

### 2.1 Cells lines

*Vero* (ATCC CCL81), HEK-293 (ATCC CRL1573) and HEK-293T cells (ATCC CRL3216) were maintained at 37°C, 5% CO_2_ in Dulbecco Modified Eagle’s Medium (DMEM, Thermo Fisher scientific, Montigny-le-Bretonneux, France) supplemented with 5% fetal bovine serum (FBS, Lonza), 1 mM sodium pyruvate, penicillin (1 U/mL)/streptomycin (1 μg/mL) and 2 mM L-glutamine. BHK-21 cells (ATCC CCL10) were maintained at 37°C in Dulbecco Modified Eagle’s Medium (DMEM, Thermo Fisher scientific) supplemented with 10% fetal bovine serum (FBS, Lonza, France) and penicillin (1 U/mL)/streptomycin (1 μg/mL). C6/36 cells (ATCC CRL1660) were maintained at 28°C, in Leibowitz L-15 Medium supplemented with 1 mM sodium pyruvate, penicillin (1 U/mL)/streptomycin (1 μg/mL), 1 mL non-essential amino acids, and 2 mM L-glutamine (Thermo Fisher Scientific, France).

### 2.2 Generation of chimeric viruses using innovative reverse genetic ISA method

The procedure is described in detail in Aubry et al. (2014). Briefly, overlapping PCR fragments generated with a high-fidelity polymerase and reconstituting the entire WNV genome of IS98, IT08 or chimeric IS98-IT08 viruses were introduced by transfection of cells sustaining efficient WNV replication.

#### 2.2.1 Preparation of PCR products for the ISA method

The complete genome, flanked at the 5′ and 3′ extremities by the human cytomegalovirus promoter (pCMV) and the hepatitis delta ribozyme, respectively, followed by the simian virus 40 polyadenylation signal [HDR/SV40p(A)], was amplified by PCR in 6 overlapping DNA fragments of approximately 2.5kb, 1.1 kb, 1.1 kb, 3.2 kb, 2.9 kb and 725 bp. The WNV IS98 strain was recovered from an infectious clone construct generated by Bahuon et al. (2012) (WNV IC-IS98). For WNV IC-IS98 and IT08, DNA fragments were obtained by RT-PCR from clarified cell supernatants. Total RNA was extracted using the MagVet Universal Isolation kit (ThermoFisher Scientific, Montigny-le-Bretonneux, France) according to the manufacturer’s instructions and amplified using the Invitrogen™ SuperScript™ IV One-Step RT-PCR System with the Platinum SuperFi DNA polymerase (ThermoFisher Scientific, Montigny-le-Bretonneux, France) to minimize PCR mutations. Amplifications were performed on an AB 7300 Real-Time PCR thermocycler (Applied Biosystems) with the following conditions: (i) 50°C for 30 min (cDNA synthesis), (ii) 94°C for 2 min (pre-denaturation), (iii) 40 amplification cycles with 94°C for 15 s–64°C for 30 s–72°C for 2 min or more – depending on the expected fragment size (1 min/kb), (iiii) final elongation at 72°C for 10 min. The size of all PCR products was verified by gel electrophoresis and PCR products were purified using PCR PureLink™ purification kit (Invitrogen, Paris, France).

PCR fragments were amplified from WNV IC-IS98 (called WN-IS98) and WNV IT08 (WN-IT08) viral genomic RNA with the following primers listed in Table 1.

**TABLE 1:** Oligonucleotides sequences used for PCR amplification in the ISA method.

N PCR fragment	WN-IS98	WN-IT08
1	Forward-AGTAGTTCGCCTGTGTGAGCTG	Same as WN-IS98
	Reverse-AGCTCTTGCCGGCTGATGTCTATG	Same as WN-IS98
2	Forward-ACGTTTCTCGCAGTTGGAGGCCCAAC	Same as WN-IS98
	Reverse-AAGAACACGACCAGAAGGCCCAAC	Same as WN-IS98
3	Forward-CCCTCGTGCAGTCACAAGTGAA	Same as WN-IS98
	Reverse-GGTGGTCATGTCCCCCTTTTTGTA	Same as WN-IS98
4	Forward-ATGCTCAGAATGGTCTGTCTCGC	Forward-TCCTGCCCTCAGTAGTTGGAT
	Reverse-CCTCTTTGCGGTACCTAGTGAAC	Reverse-CCTCTTTGCGGTACCTAGTGAAC
5	Forward-TGAACGCAACAACTGCCATCG	Forward-ATTGGACTCTGCCACATCATGCG
	Reverse-CCGGCCTGACTTTTCTC	Reverse-CCGGCCTGACTTCCTCCTTAAA
6	Forward-CCGAGCCACGTGGGCAGAAAAYA	Same as WN-IS98
	Reverse-AGATCCTGTGTTCTCGCACCACC	Same as WN-IS98

For each genome (WN-IS98 and WN-IT08) six PCR fragments were generated.

#### 2.2.2 Cells transfection

Cell transfection was performed as previously described by Driouich et al. (2019). Briefly, 1 day before transfection, an equal number of HEK-293T and BHK-21 cells were plated in 96-well plates at the final density of 2 × 10^4^ cells/well (1 × 10^4^ HEK-293T cells + 1 × 10^4^ BHK-21 cells). Transfection was performed using the Lipofectamine 3000 reagent (Invitrogen, Paris, France) and optiMEM (Gibco, ThermoFisher Scientific, Montigny-le-Bretonneux, France). Cells in each well were transfected with a 100 ng aliquot of an equimolar mix of the 8 DNA fragments (6 for the viral genome, 1 for pCMV and 1 for HDR/SV40p(A) ribozyme as previously described (Aubry et al., 2014). Each transfection was performed in 9 wells to ensure the production of the chimeric construct in at least one well. Transfected cells were incubated at 37°C, 5% CO_2_ for 24 h. At 24 h post-transfection, cell supernatants were replaced by 100 μL of fresh DMEM medium supplemented with 5% fetal bovine serum (FBS, Lonza, France), 1 mM sodium pyruvate, penicillin (1 U/mL)/streptomycin (1 μg/mL) and 2 mM L-glutamine. Five days after transfection, cell supernatants were collected and then passaged once on confluent *Vero* cells plated in 96-well plates. After an incubation period of 5 h, cells were washed with PBS 1X and 100 μL of fresh medium was added per well. Cell supernatants were passaged twice on confluent *Vero* cells. Between each passage, cell supernatants were diluted 1/1000 to ensure that infectious viruses were produced. The resulting virus stocks, obtained after a total of 3 passages on *Vero* cells, were used to confirm the production of infectious particles by RT-qPCR, TCID_50_ titration and whole-genome next-generation sequencing.

### 2.3 Next-generation sequencing of chimeric viruses

Next-generation sequencing was performed using Ion PGM Instrument with the Torrent Suite 5.12 Software (ThermoFisher Scientific, France). Whole genome sequencing analyses were performed using the SISPA method initially developed for sequencing on Illumina platforms and further adapted to IonTorrent workflows (Gil et al., 2021). Briefly, the sequencing consisted of three steps: (i) library preparation using random amplification and PCR addition of library-adapters, (ii) library sizing quantification and quality assessment, and (iii) sequencing. Library preparation included a first step of random amplification. Briefly, a reverse transcription produced cDNA from RNA molecules using TagE_8N_HPLC random oligonucleotides (5′-CATCACATAGGCGTCCGCTGNNNNNNNN-3′) using the RevertAid First Strand cDNA Synthesis kit (Fisher Scientific, Les Ulis, France). Reverse transcription was followed by Klenow polymerization (Fisher Scientific, Les Ulis, France) and high-fidelity PCR with the Phusion High-Fidelity DNA Polymerase kit (Fisher Scientific, France). 4 PCR reactions per sample were performed to generate sufficient amplicons. The PCR mix (50 μl) consisted of 0.2 μM primer (TagEshort 5′-CATCACATAGGCGTCCGCTG-3′), 0.2 mM dNTPs, 1 × Phusion HF buffer, 0.5U of Phusion DNA polymerase and 5 μl of dsDNA obtained after Klenow polymerization. PCR parameters were as follows: 30 s at 98°C, 32 cycles of 10 s at 98°C, 20 s at 65°C, 60 s at 72°C, followed by a final elongation of 10 min at 72°C. PCR reactions from a given sample were pooled and purified with the NucleoSpin gel and PCR Clean-up (Macherey-Nagel, Hoerdt, France) following the manufacturer’s instructions. Amplicons were eluted in 35 μl of ultra-pure water.

IonTorrent adapters (LigA_[n501-513], 5′-CCATCTCATCCC TGCGTGTCTCCGACT-3′ bound to 8-mer barcodes for the multiplexing of sequencing reactions) were added by PCR to TagE-flanked amplified dsDNA with the Ion Plus Fragment Library kit (ThermoFisher Scientific, Montigny-le-Bretonneux, France); the PCR mix consisted of 50 μL of Platinum PCR Supermix High Fidelity, 0.1 μM primers (LigA_[n501-513]-TagE and Lig2P1-TagE 5′-CCACTACGCCTCCGCTTTCCTCTCTA TGGGCAGTCGGTGATCATCACATAGGCGTCCGCTG-3′) and 7 μl of dsDNA. 4 PCR reactions per sample were carried out as follows: 5 min at 95°C, 8 cycles of 15 s at 95°C, 15 s at 58°C, 60 s at 70°C. PCR products from a given sample were pooled and purified with Agencourt AMPure XP (Fisher Scientific, France) following the manufacturer’s instructions. Amplicons bound to IonTorrent adapters were eluted in 35 μl of ultra-pure water.

Library sizing was performed by gel electrophoresis with eGel Size-Select Agarose (Thermofisher Scientific, Montigny-le-Bretonneux, France) following the manufacturer’s instructions. Bands of approximately 200 bp were excised from the agarose gel. Library concentrations were quantified and their quality assessed on the Bioanalyzer Instrument with an Agilent High Sensitivity PCR kit (Agilent Technologies, Waldbronn, Germany) following the manufacturer’s instructions.

Libraries were multiplexed at equimolar ratios. The Ion OneTouch™ 2 System was used to prepare enriched, template-positive Ion PGM™ Hi-Q™ Ion Sphere™ Particles (ISPs) bound to insert libraries following the manufacturer’s instructions (Thermofisher Scientific, Montigny-le-Bretonneux, France). Libraries were sequenced using the Ion PGM™ Hi-Q™ Sequencing Kit and Ion 316™ Chip v2 BC (Thermofisher Scientific, Montigny-le-Bretonneux, France) on an IonTorrent PGM instrument (Thermofisher Scientific, Montigny-le-Bretonneux, France).

Sequencing data were processed using a custom viral bioinformatics pipeline. Viral reads were identified and extracted through read mapping to the WN-IS98 genome (AF481864) using bwa-mem2 v2.0 (Vasimuddin et al., 2019). Adaptors, primer sequences, and low-quality bases (phred score threshold of 20) were trimmed from the raw reads, followed by removal of duplicate reads. Filtered datasets were assembled using SPAdes v3.15.5 (Prjibelski et al., 2020) using the default multiple kmer lengths and settings specific for Ion Torrent datasets. Resulting contigs were compared to the Pfam conserved domain DB- using blastx (Altschul et al., 1990). The shared regions within the chimeric virus reconstruction is visualized through a Hive Plot built with d3.js (Bostock et al., 2011).

### 2.4 WNV plaque phenotype

**FIGURE 1:**
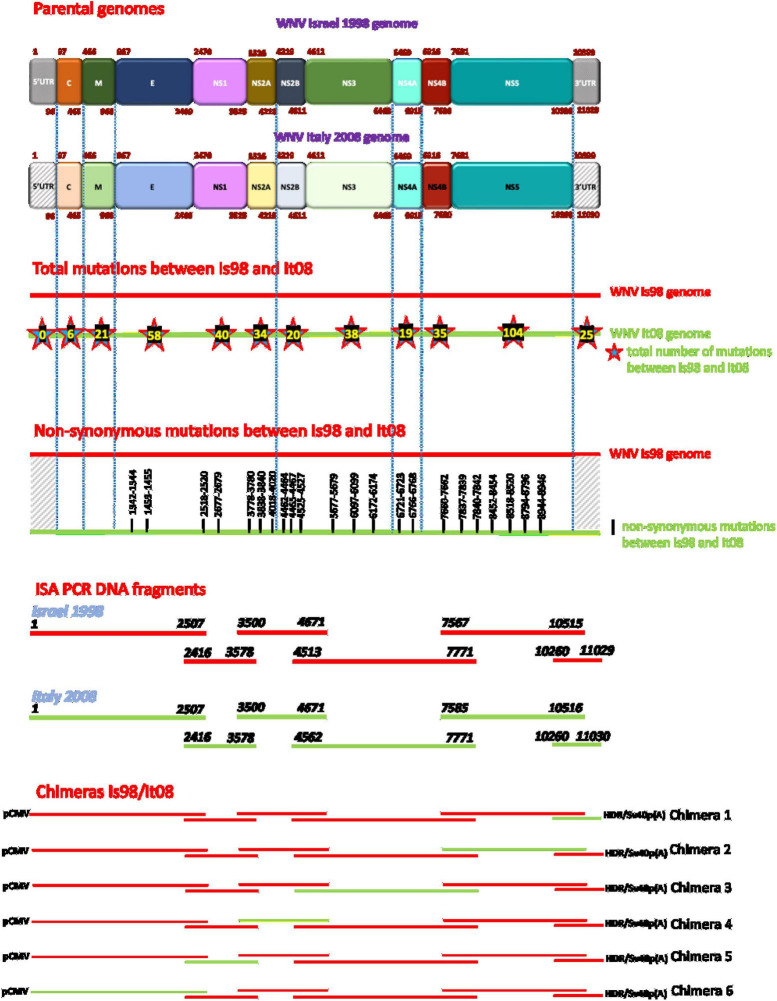
Schematic representation of chimeric constructs generated by the ISA method. Red lines represent WN-IS98 genome or corresponding PCR products. Green lines represent WN-IT08 genome or corresponding PCR products. The total number of synonymous and non-synonymous mutations between the two strains are reported inside stars on WN-IT08 genome. The exact start and end nucleotide positions are indicated for each ISA PCR DNA fragments. Chimeras are flanked at 5′ and 3′ ends by pCMV promoter and HDR/SV40p(A), respectively.

*Vero* cells were seeded in 6-well plates and infected with parental WN-IS98 and WN-IT08 strains and the six chimeric viruses (Figure 1) at 100 PFU for 1 h 30 at 37°C, 5% CO_2_, after which time media containing the virus was removed. Cells were overlaid with a semi-solid medium containing 2.4% Avicel, MEM 2X (ThermoFisher Scientific, Montigny-le-Bretonneux, France), 2.5% FBS (Lonza, France), 1% penicillin/streptomycin (ThermoFisher scientific, Montigny-le-Bretonneux, France) and 1% sodium pyruvate and incubated for 3 days at 37°C, 5% CO_2_. The overlay was removed; cells were washed twice in PBS 1X and fixed with 4% paraformaldehyde and stained with 0.4% crystal violet for 24 h at room temperature.

### 2.5 Viral growth kinetics

*Vero* cells were seeded in 24-well plates and infected with parental WN-IS98 and WN-IT08 strains and the six chimeric viruses at MOI 0.1 in DMEM supplemented with 2% FBS. The cells were incubated at 37°C, 5% CO_2_ and cell supernatants were harvested at the indicated times post-infection (17 h, 24 h, 48 h, and 72 h). Number of genome copies and infectious titers were determined as described below for all time points.

### 2.6 Quantitative RT-PCR

Total RNA was extracted using the MagVet™ Universal Isolation kit (ThermoFisher scientific, Montigny-le-Bretonneux, France) according to the manufacturer’s instructions. Reverse transcription and amplification were performed using the AgPath-ID One-Step RTPCR Kit (Applied Biosystems) as previously described in Linke et al. (2007). WNV amplification was achieved using the following specific primers (final concentration of 0.4 μM) WNproC-10 5′-CCTGTGTGAGCTGACAAACTTAGT-3′ and WNproC-132 5′-GCGTTTTAGCATATTGACAGCC-3′ and the probe (final concentration of 0.2 μM) 5′–FAM -CCTGGTTT CTTAGACATCGAGATCT–Tamra–3′. The housekeeping gene β -Actin was amplified using the primers (final concentration of 0.4 μM) ACTB-966 5′-CAGCACAATGAAGATCAAGATCATC-3′ and ACTB-1096 5′-CGGACTCATCGTACTCCTGCTT-3′ and the probe (final concentration of 0.2 μM) 5′-VIC-TCGCTGTCCACCTTCCAGCAGATGT-TAMRA-3′ (Toussaint et al., 2007). RT-PCR were run in an AB 7300 Real-Time PCR system (Applied Biosystems, Villebon sur Yvette, France) using the following program: 45°C for 10 min, 95°C for 10 min followed by 40 cycles of 95°C for 15 s and 60°C for 60 s. Genome copies per microliter were calculated based on the threshold cycle (CT) values of a standard curve generated using the standard RNA of WNV and normalized using the β-actin signal.

### 2.7 Infectious titer determinationby TCID_50_

TCID_50_ was determined using 96-well cell culture plates seeded 1 day prior to infection with *Vero* cells at a density of 2.10^4^ cells/well. Briefly, virus stocks were 10-fold serially diluted in DMEM and 50 μL of each dilution were inoculated into each well in quadruplicate. Plates were incubated for 3 days at 37°C, 5% CO_2_ and 50% TCID_50_ was determined by counting wells displaying viral cytopathic effect (CPE) using the methodology described by Reed and Munch.

### 2.8 Virulence in mice

Seven-week-old BALB/cByJ mice (Charles River Laboratories, L’Arbresle, France) were housed in an environmentally controlled room under biosafety level 3 conditions and were given food and water *ad libitum*. BALB/cByJ have long been used for studying WNV pathology and pathogenesis in mammals; they are highly susceptible to WNV clinical disease, due to impaired innate immunity associated with the absence of OAS1B expression (a premature STOP codon in the OAS1B gene is found in virtually all inbred mouse strains) and develop neurological signs and lesions mimicking the ones observed in humans and horses (Basset et al., 2020).

Groups of five mice were inoculated intraperitoneally with 10 PFU of WN-IS98, WN-IT08, chimera IS98-3′NS4B/NS5 or IS98-3′NS2B/NS3/NS4A/NS4B prepared in DMEM or with DMEM alone as a negative control. Every day, animals were weighed and monitored for the duration of the study (12 days) to detect the onset of signs of illness or suffering. Mice were euthanized by cervical dislocation when the end point was reached, that is, when at least two of the following clinical signs were observed: weight loss greater than 15%, anorexia, ruffled hair, curved back, loss of balance, paresis. The presence of viral RNA was confirmed by RT-qPCR in blood collected at 3-day pi (dpi) and in brain, heart and lung tissues, recovered shortly after their death and homogenized using the FastPrep Instrument (MP Biomedicals, Illkirch, France).

BALB/cByJ mice were housed at ANSES animal facilities (Maisons-Alfort, Paris). Work on animals was performed in compliance with French and European regulations on welfare and protection of animals used for scientific purposes (EC Directive 2010/63, French Law 2013-118, February 6th, 2013). All experiments were approved by the joint Anses-UPEC-Alfort Veterinary School ethics committee under the permit number 2022-11-15-11.

Biosecurity measures for the biocontainment of parental and chimeric viruses generated in this study have been reviewed and validated by the French Ministry of Higher Education, Research and Innovation under the DUO 10537.

### 2.9 Vector competence of *Cx. pipiens* mosquitoes

Infections of *Cx. pipiens* mosquitoes were performed at the Institut Pasteur, Paris, France. Briefly, 5- to 7-day-old female mosquitoes were transferred into plastic boxes and starved for 24 h in a biosafety level 3 insectarium before being orally infected with WN-IS98, WN-IT08, chimera IS98-3′UTR or IS98-3′NS4B/NS5 provided in a blood-meal corresponding to washed rabbit erythrocytes (2/3) mixed with a viral sample (1/3) at a final concentration of 10^7^ TCID_50_/mL. Adenosine triphosphate (ATP) (Sigma-Aldrich) was added as a phagostimulant at a final concentration of 5 × 10^–3^ M. Fully engorged females were maintained in cardboard boxes, at 28°C and 80% humidity for 14 days with a 12 L: 12 D circadian cycle and then processed for saliva collection (Amraoui et al., 2016). Batches of around twenty female mosquitoes were dissected at 14 days post-infection after cold anesthesia. Briefly, legs and wings of each mosquito were removed, and the proboscis was inserted into a 20 μL tip containing 5 μL of FBS for saliva collection. Head/thorax and body were separated from each mosquito and ground individually in DMEM (ThermoFisher Scientific, Montigny-le-Bretonneux, France). For each mosquito, abdomen was removed and processed alone to characterize infection. Head and thorax were processed together to characterize dissemination (crossing the intestinal barrier) and saliva was collected to characterize transmission. Each mosquito sample (abdomen, head and thorax, saliva) was analyzed separately. Abdomen and head/thorax were grinded in 500 uL of medium. The infection rate and dissemination efficiency were assessed using RT-qPCR detection on body and head, respectively, whereas transmission efficiency was performed using TCID_50_ viral titration on saliva. Infection rate corresponds to the percentage of mosquitoes presenting positive Ct after abdomen homogenization, compared to total mosquitoes dissected. Dissemination efficiency corresponds to the percentage of mosquitoes presenting positive Ct after head/thorax homogenization, compared to the total of mosquitoes positive for infection. Transmission efficiency corresponds to the percentage of positive saliva (evaluated by TCID_50_ assay) compared to total mosquitoes positive for infection.

### 2.10 Statistical analysis

All statistical analyses were performed using GraphPad Prism software and the appropriated Kruskal-Wallis or Fisher’s exact tests.

## 3 Results

### 3.1 Production of chimeric viruses between WN-IS98 and WN-IT08

**FIGURE 2:**
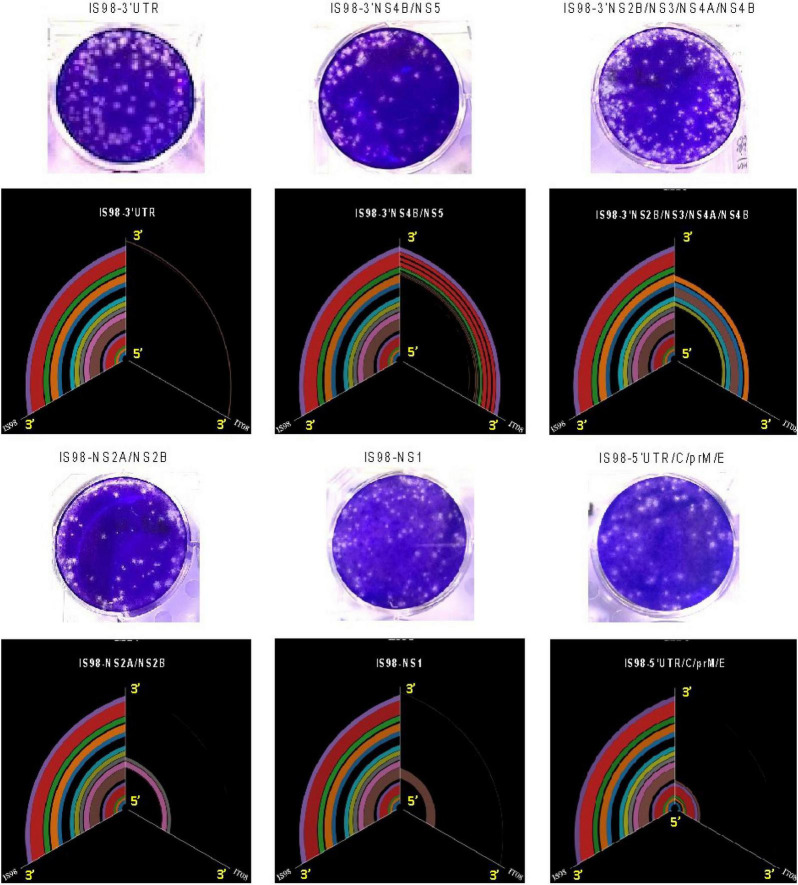
Hive plot representation of the sequence of the chimeric constructs. The middle axis represents the chimeric genome, the left axis the WN-IS98 genome – the line between the two represents the shared genomic regions, based on blastx alignment on the pfam conserved domain DB, the right axis is the WN-IT08 strain and lines between the middle and right axis represent shared regions between WN-IT08 and the chimeras.

Six chimeric viruses constructed by inserting different genomic fragments (3′UTR, 3′NS4B/NS5, 3′NS2B/NS3/NS4A/NS4B, NS2A/NS2B, NS1, 5′UTR/C/prM/E) from the low virulent WN-IT08 into the WN-IS98 genome were obtained by using ISA. Next-generation sequencing confirmed that chimeric constructs had indeed been obtained. Briefly, Figure 2 corresponds to the hive plot representation of the sequencing data obtained for the 6 WN-IS98/IT08 chimeras generated. For each chimera, three axes are represented. The one on the left corresponds to WN-IS98 genome, the right axis corresponds to WN-IT08 genome and the other corresponds to the chimera sequence. Each colored curve represents genomic similarities between the parental viruses and the chimeric construct. Here, we confirm that chimeras comprised the WN-IS98 backbone with WN-IT08-derived fragments corresponding to the 3′UTR (IS98-3′UTR), 3′NS4B and NS5 (IS98-3′NS4B/NS5), 3′NS2B, NS3, NS4A and NS4B (IS98-3′NS2B/NS3/NS4A/NS4B), NS2A and NS2B (IS98-NS2A/NS2B), NS1 (IS98-NS1), and finally the 5′UTR, C, prM and E (IS98-5′UTR/C/prM/E). The sequence of WNV IC-IS98 and derived chimeras differed from the reference IS-98 STD strain (Genbank accession number AF481864.1) by 7 non-synonymous mutations: NS1-N17S; NS2A-R165G; NS2B-G82D; NS2B-E83G, NS3-P496L, NS3-E521D and NS5-N280K. These mutations probably reflect sequencing errors of the reference IS-98 STD strain or mutations having occurred during isolation and the first passages of the IS-98 STD strain, since the sequence of WNV IC-IS98 was determined to be identical to the parental WN-IS98 isolate used in our experimental assays. Of note, the replicative properties of IC-IS98 bearing these non-synonymous mutations were indistinguishable from those of the parental WN-IS98 isolate *in vitro* or *in vivo* (Bahuon et al., 2012).

Of note, a few mutations were introduced during the ISA procedure. Seven non-synonymous mutations have been identified by the sequencing of the complete genomes of the chimeras, that could have resulted from the amplification of naturally-occurring mutations in WN-IT08 and WN-IS98 isolates (quasi-species) or from the generation of errors during the amplification step. These mutations are: NS1-N207I (found on the chimera IS98-3′NS2B/NS3/NS4A/NS4B), NS3-R250K (found on chimera IS98-NS2A/NS2B), NS4B-C120F (found on chimera IS98-3′UTR), NS5-S54P (found on chimera IS98-3′NS2B/NS3/NS4A/NS4B and IS98-NS2A/NS2B), NS5-P431Q (found on chimera IS98-NS1), NS5-L432I (found on chimera IS98-5′UTR/C/prM/E) and NS5-V788I (found on chimera IS98-NS2A/NS2B).

In conclusion, six WN-IS98/WN-IT08 chimeric viruses were produced, with genomic sequences sharing more than 99.98% nt identity with the expected sequence.

### 3.2 Multiplication kinetics of the parental wild type (WT) isolates and chimeras in mammalian cell cultures

**FIGURE 3:**
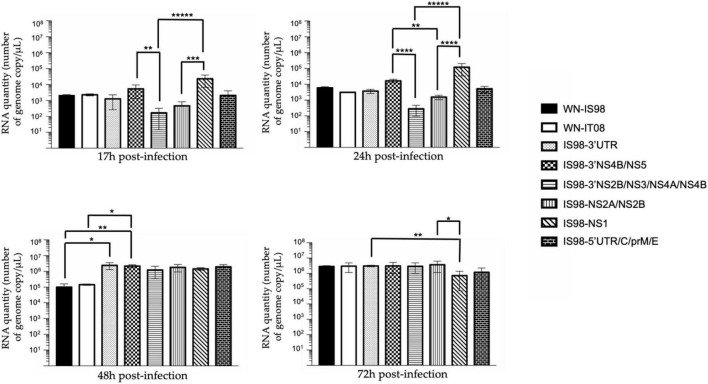
RT-qPCR analysis at 17 h, 24 h, 48 h, 72 h post-infection of parental strains and chimeras. *Vero* cells were infected with the indicated virus at multiplicity of infection (MOI) of 0.1. At the indicated time, cell culture supernatants were collected and analyzed by RT-qPCR. Number of genome copy/μL were quantified. Group statistical comparisons were performed by the non-parametrical Kruskal-Wallis test. Paired statistical comparisons were performed by Dunn’s analysis. Results represent the mean of duplicate experiments, and in each experiment each point was performed in triplicate. The error bar indicates the standard deviation (SD) of duplicate measures. *0.05 < *P* < 0.1; **0.01 < *P* < 0.05; ***0.001 < *P* < 0.01; ****P < 0.001; *****P < 0.0001.

**FIGURE 4:**
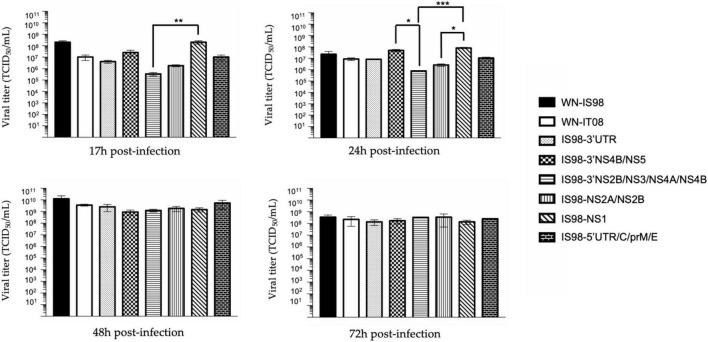
TCID_50_ quantification at 17 h, 24 h, 48 h, and 72 h post-infection of parental strains and chimeras. *Vero* cells were infected with the indicate virus at multiplicity of infection (MOI) of 0.1. At the indicated time, cell culture supernatants were collected and analyzed by TCID_50_ quantification. Infectious virus titers were expressed in TCID_50_/mL. Each sample was analyzed in triplicate. Group statistical comparisons were performed by the non-parametrical Kruskal-Wallis test. Paired statistical comparisons were performed by Dunn’s analysis. Results are represented as the mean of duplicate experiments, and in each experiment each point was performed in triplicate. The error bar indicates the standard error of the mean (SEM) of triplicate measures. *0.05 < *P* < 0.1; **0.01 < *P* < 0.05; ***0.001 < *P* < 0.01.

Replication kinetics were performed on *Vero* cells to compare the replicative fitness of each chimeric construct and parental strain in susceptible mammalian cells. *Vero* cells were infected at MOI 0.1 and cell culture supernatants were harvested at 17 h, 24 h, 48 h, and 72 h pi. Amounts of viral RNA and infectious particles were measured using RT-qPCR assay (number of genome copy/μL) and TCID_50_, respectively. Replication kinetics of parental viruses were similar (Figure 3). Differences, however, were observed between chimeras, especially at 17 h and 24 h p.i. Of note, total viral RNA was significantly lower for IS98-3′NS2B/NS3/NS4A/NS4B than for IS98-3′NS4B/NS5 (*p* < *0.05*) or IS98-NS1 (*p* < *0.0001*) at 17 h and 24 h p.i only. This result was confirmed by a significantly lower infectious titer for IS98-3′NS2B/NS3/NS4A/NS4B than for IS98-NS1 at 17 h and 24 h p.i., but the difference between IS98-NS2B/NS3/NS4A/NS4B and IS98-3′NS4B/NS5 at 17 h p.i was not statistically significant (Figure 4).

These results suggest that the replication of IS98-3′NS2B/NS3/NS4A/NS4B is decreased at early time points of infection before reaching a similar level at later time points (48 h and 72 h p.i.).

The same observations were made when considering IS98-NS2A/NS2B and IS98-NS1. The quantity of total viral RNA was lower for IS98-NS2A/NS2B than for IS98-NS1 (*p* < *0.01*) at 17 h and at 24 h p.i. (*p* < *0.0001*) (Figure 3). Viral infectious titers confirmed these results only at 24 h p.i (>10^6^ TCID_50_/mL for IS98-NS2A/NS2B and >10^8^ TCID_50_/mL for IS98-NS1) (Figure 4). Surprisingly, quantification of total RNA at 48 h showed impaired replication of WN-IS98 and WN-IT08 in comparison with IS98-3′UTR and IS98-3′NS4B/NS5.

In sum, two chimeras demonstrated impaired replication kinetics in *Vero* cells, namely, IS98-3′NS2B/NS3/NS4A/NS4B and IS98-NS2A/NS2B, in comparison with chimeras IS98-NS1 and IS98-3′NS4B/NS5.

### 3.3 Replication of parental and chimeric viruses in BALB/cByJ mice

*In vivo* experiments performed in BALB/cByJ mice, which are highly susceptible to WNV, allowed us to characterize WNV pathogenicity regarding viremia, virus dissemination in peripheral tissues (data not shown), neuroinvasiveness and virulence. BALB/cByJ mice were infected by WN-IS98 and WN-IT08 strains and by chimeras IS98-3′NS4B/NS5 and IS98-3′NS2B/NS3/NS4A/NS4B, this latter having demonstrated decreased replication in comparison with IS98-3′NS4B/NS5 (control chimera) at early time points in mammalian cells. We hypothesized that such impaired replication of IS98-3′NS2B/NS3/NS4A/NS4B could affect early replication in mice and consequently decrease viremia.

**FIGURE 5:**
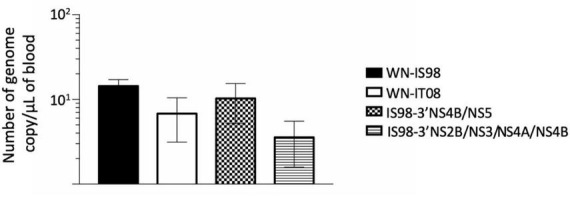
Viremia 3 days after intraperitoneal inoculation of female BALB/cByJ mice with 10 PFU of WNV IS98, WNV IT08 and chimeras IS98-3′NS4B/NS5 and IS98-3′NS2B/NS3/NS4A/NS4B. Viral RNA load in blood was quantified by RT-qPCR. The error bar indicates standard error of the mean (SEM).

As expected, a peak of viremia was reached at 3 days post-infection (data not shown). No significant differences were observed between the tested viruses. Nevertheless, IS98-3′NS2B/NS3/NS4A/NS4B tended to induce lower viremia (<10^1^ RNA copy/μL) than WN-IS98 and IS98-3′NS4B/NS5 (Figure 5).

**FIGURE 6:**
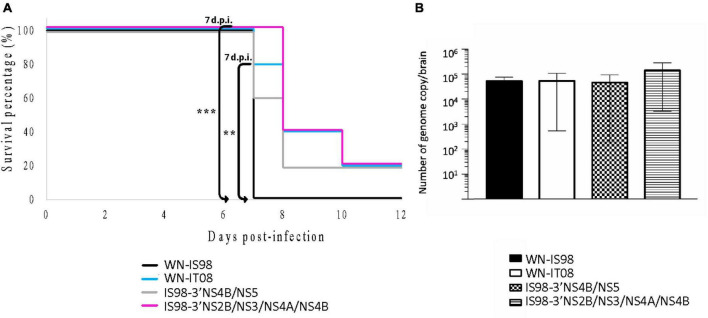
Neuroinvasive properties and neurovirulence of WN-IS98, WN-IT08 and their chimeras in 2-week-old female BALB/cByJ mice. Mice were infected intraperitoneally with 10 PFU and monitored for 12 dpi. **(A)** Survival growth. **(B)** Genome quantification in the brain of dead mice assessed by RT-qPCR. The error bar indicates standard error of the mean (SEM). **0.01 < *P* < 0.05; ***0.001 < *P* < 0.01.

BALB/cByJ were monitored 12 days after i.p. inoculation. All mice infected with the parental strain WN-IS98 died at day 7 p.i., whereas only one mouse infected with WN-IT08 and 2 mice infected with IS98-3′NS4B/NS5 died at the same day p.i. Delayed death during infection by IS98-3′NS2B/NS3/NS4A/NS4B became evident on day 8 p.i. Kruskal-Wallis analysis at 7 days p.i. showed a significant difference in mortality between IS98-3′NS2B/NS3/NS4A/NS4B and WN-IS98 (*p* < *0.01*) and between WN-IT08 and WN-IS98 (*p* < *0.05*). No other differences were observed in mortality (Figure 6A).

Since WNV is a neurotropic virus, neuroinvasion and viral load in the CNS are relevant parameters to evaluate in the course of WNV infection. Total RNA in the brain was quantified by RT-qPCR. Although no statistical differences were observed in the level of total RNA (Figure 6B), WNV was detected in the brains of animals belonging to all four groups. Although the quantity of total RNA in brain post-mortem was comparable for all four viruses, WN-IT08, IS98-3′NS4B/NS5 and IS98-3′NS2B/NS3/NS4A/NS4B seemed to induce delayed mortality in comparison with WN-IS98.

### 3.4 Multiplication kinetics of the parental wild type (WT) isolates and chimeras in insect cell cultures (C6/36)

**FIGURE 7:**
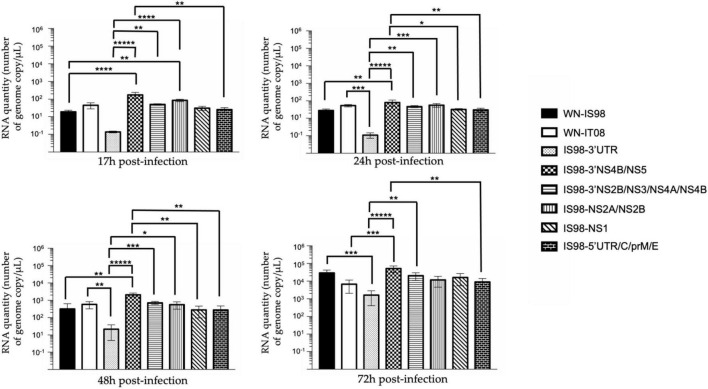
RT-qPCR analysis at 17 h, 24 h, 48 h, 72 h post-infection of parental strains and chimeras. C6/36 cells were infected with the indicated virus at multiplicity of infection (MOI) of 0.1. At the indicated time, cell culture supernatants were collected and analyzed by RT-qPCR. Number of genome copies/μL was quantified. Group statistical comparisons were performed by the non-parametrical Kruskal-Wallis test. Paired statistical comparisons were performed by Dunn’s analysis. Each mean representation results from duplicate experiments, and in each experiment each point was performed in triplicate. The error bar indicates standard deviations (SD). *0.05 < *P* < 0.1; **0.01 < *P* < 0.05; ***0.001 < *P* < 0.01; ****P < 0.001; *****P < 0.0001.

Replication kinetics were assessed on the *Aedes albopictus* mosquito cell line, C6/36. In contrast to results obtained in *Vero* cells showing no impairment in replication of parental WNV isolates, the level of total viral RNA was significantly lower for the parental strain WN-IS98 than the IS98-3′NS4B/NS5 chimera at 17 h (*p* < *0.0001*), 24 h (*p* < *0.05*) and 48 h (*p* < *0.05*) p.i. (Figure 7). Moreover, the quantity of total RNA seemed to be significantly reduced for IS98-3′UTR than for the parental strain WN-IT08 at 24 h (*p* < *0.001*), 48 h (*p* < *0.05*) p.i. and compared to WN-IS98 at 72 h p.i. (*p* < *0.01*). IS98-3′UTR is the chimeric construct that demonstrated the lower RNA quantity when compared with other chimeras, especially with IS98-3′NS4B/NS5 at 17 h (*p* < *0.0001*), 24 h (*p* < *0.0001*), 48 h (*p* < *0.0001*) and even 72 h p.i. (*p* < *0.0001*). Moreover, IS98-3′NS4B/NS5 presented significantly higher viral RNA copies than IS98-5′UTR/C/prM/E at 17 h (*p* < *0.05*), 24 h (*p* < *0.05*), 48 h (*p* < *0.05*) and 72 h (*p* < *0.05*).

**FIGURE 8:**
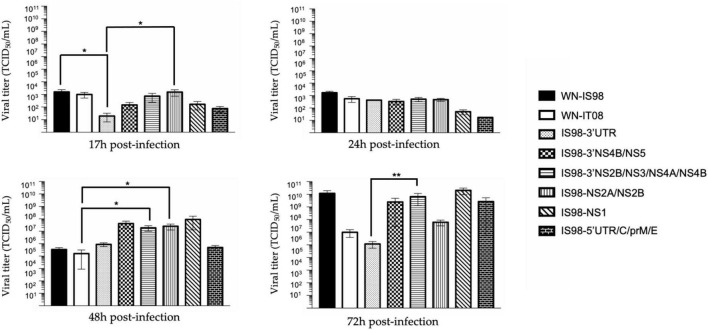
TCID_50_ quantification at 17 h, 24 h, 48 h, and 72 h post-infection of parental strains and chimeras. C6/36 cells were infected with the indicated virus at multiplicity of infection (MOI) of 0.1. At the indicated time, cell culture supernatant was collected and analyzed by TCID_50_ quantification. Viral quantities of infectious virus are expressed in TCID_50_/mL. Each sample was analyzed in triplicate. Group statistical comparisons are performed by the non-parametrical Kruskal-Wallis test. Paired statistical comparisons are performed by Dunn’s analysis. Results are represented as means od duplicate experiments, and in each experiment each point was performed in triplicate. The error bar indicates standard error of the mean (SEM) for each triplicate. *0.05 < *P* < 0.1; **0.01 < *P* < 0.05.

Cell supernatants of C6/36 mosquito cells infected at 0.1 MOI were collected at 17 h, 24 h, 48 h and 72 h p.i. IS98-3′UTR showed decreased replication in comparison with IS98-NS2A/NS2B and WN-IS98 at 17 h p.i. (*p* < *0.1*) and with IS98-3′NS2B/NS3/NS4A/NS4B at 72 h p.i. (*p* < *0.05*) (Figure 8). In conclusion, the insertion of the 3′UTR of the WN-IT08 strain in the WN-IS98 genome impaired IS98-3′UTR replication in C6/36 mosquito cells at most time points (17–72 h p.i.).

### 3.5 Replication of parental and chimeric viruses in *Cx. pipiens* mosquitoes

Compared to *in vitro* analyses, *in vivo* testing in mosquitoes allows evaluation of vector competence, regarding infection rate (IR), dissemination efficiency (DE) and transmission efficiency (TE).

In order to assess the differential ability of parental and chimeric viruses to cross the midgut barrier and spread in *Cx. pipiens* mosquitoes, the IR, DE, and TE were determined for parental strains WN-IS98 and WN-IT08, as well as for chimeras IS98-3′UTR and IS98-3′NS4B/NS5 (control chimera). IS98-3′UTR has been shown to replicate less efficiently in mosquito cells, and we hypothesized that such properties could decrease IR, DE or TE in *Cx. pipiens* mosquitoes. While IR reflects the infection rate of engorged female mosquitoes among tested individuals, DE is the proportion of infected females in which WNV was able to cross the midgut barrier and penetrate the mosquito haemocoel. Finally, the ability of each tested virus to reach the salivary glands was evaluated by determining the transmission efficiency (TE), which corresponds to the proportion of female mosquitoes that secrete infectious saliva among tested specimens.

**FIGURE 9:**
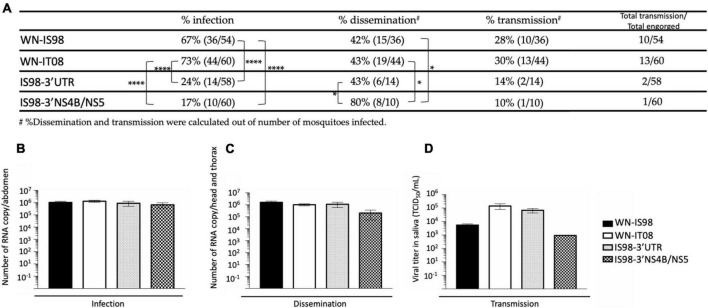
Infection, dissemination, and transmission rates after a blood meal with 10^7^ TCID_50_/mL of WN-IS98, WN-IT08, IS98-3′UTR and IS98-3′NS4B/NS5. **(A)** Percentage of mosquitoes presenting an infection, dissemination, or transmission. **(B)** Viral titer in abdomen assessed by RT-qPCR. **(C)** Viral titer in head and thorax assessed by RT-qPCR. **(D)** Viral titer in saliva assessed by TCID_50_ titration. The error bar indicates standard error of the mean (SEM). *0.05 < *P* < 0.1; ****P < 0.001.

The IRs were significantly higher for parental strains than for chimeric viruses IS98-3′UTR and IS98-3′NS4B/NS5 (Figure 9A).

Infection of *Cx. pipiens* was higher for WN-IS98 and WN-IT08 (67 and 73%, respectively) (Fisher’s exact test) than for IS98-3′UTR (24%) (*p* < *0.001*) and IS98-3′NS4B/NS5 (17%) (*p* < *0.001*). No significant differences, however, were observed between parental viruses or between the chimeras as regards infection rates. Concerning the percentage of dissemination, significant differences were observed between IS98-3′UTR (43%) and IS98-3′NS4B/NS5 (80%) and between IS98-3′NS4B/NS5 (80%), WN-IS98 (42%) and WN-IT08 (43%). No significant differences in transmission, however, were observed among the four viruses, even though WN-IS98 and WN-IT08 tended to be more easily identified in saliva than IS98-3′UTR and IS98-3′NS4B/NS5 (Figure 9A). Similar viral RNA loads, between 10^5^ and 10^6^ RNA copies, were identified in abdomens (Figure 9B), heads and thorax (Figure 9C) after infection with parental and chimeric viruses. Although not statistically significant, differences in infectious viral titers in the saliva of infected mosquitoes were reported, with infectious titers for WN-IT08 and IS98-3′UTR (about 10^5^ TCID_50_/mL) being at least 10 times higher than those of WN-IS98 and IS98-3′NS4B/NS5 (about 10^3^ TCID_50_/mL) (Figure 9D).

In conclusion, the transmission risk of IS98-3′UTR by *Cx pipiens* mosquitoes was severely reduced (2/58, 3.4%), in comparison with WN-IS98 (10/54, 18.5%) and WN-It08 (13/60, 21.7%).

## 4 Discussion

Infectious subgenomic amplicons reverse genetics was successfully adapted to two lineage 1 Mediterranean strains of varying virulence, WN-IS98 and WN-IT08. Along with experimental infection of model hosts for WNV circulation in Europe, namely, *Cx. pipiens* mosquitoes and BALB/cByJ mice as a proxy for WNV infection in mammals, the method permitted identification of the molecular determinants of WNV virulence and pathogenicity and exploration of the mechanisms responsible for outbreaks of varying severity.

We recently identified the NS4A/NS4B/5’NS5 region of the viral genome as being implicated in increased bird mortality during Mediterranean WNV outbreaks (Fiacre et al., 2023). Here, we provide information on the role of other NS and structural proteins of WNV-IS98 and IT08 as virulence factors in mammalian hosts, as well as on factors modulating virus transmission in mosquito vectors.

Six chimeric viruses were constructed involving exchanges between WNV-IS98 and IT08, such that either structural (C, prM, E), non-structural (NS) or untranslated regions (UTR) of the WN-IS98 genome were replaced by the cognate regions of the IT08 strain. Chimeras were generated using the versatile infectious subgenomic amplicons (ISA) reverse genetic technology (Aubry et al., 2015). The ISA method avoids constraints associated with the use of infectious bacterial clones such as the propagation of potentially toxic cDNA copies of the viral genome in bacteria. Comparison of the replicative fitness of parental and chimeric viruses were carried out in mammalian and mosquito cell culture models, and virulence and viral transmission were assessed *in vivo* in mice and in *Cx. pipiens* mosquitoes, respectively.

Thirty-one mutations had been previously shown to be implicated in WNV virulence in mammalian models, including E-159, NS3-249 and NS1-175, but only a few of them, such as NS3-483, NS1-175 and NS1-207 (Fiacre et al., 2020), have been identified as molecular determinants of virus replication in mosquitos. Consequently, among the molecular markers implicated in the West Nile transmission cycle, those found not only in mammalian and avian hosts, but also in mosquito vectors, require further characterization.

We first investigated the viral fitness and pathogenicity of chimeric and parental viruses *in vitro* in *Vero* cells and *in vivo* in BALB/cByJByJ mice, respectively. Our results were indicative of a delayed replication of IS98-3′NS2B/NS3/NS4A/NS4B *in vitro*, a finding confirmed by an attenuated phenotype *in vivo*. Indeed, viral RNA loads and infectious viral titers were significantly lower than those of IS98-NS5 and IS98-NS1 at 17 h and 24 h p.i. (Figures 8, 9). Surprisingly, reduced virus replication of IS98-3′NS2B/NS3/NS4A/NS4B was no longer observed from 48 h p.i onward. Such delayed replication of IS98-3′NS2B/NS3/NS4A/NS4B may have an impact on early stages of infection and therefore on the capacity for spread and potential for neuroinvasion of IS98-3′NS2B/NS3/NS4A/NS4B in mice. Indeed, WNV spread in model mice is intimately linked to the magnitude of viremia at early time points after peripheral virus inoculation. *In vivo*, viremia quantified at day 3 p.i tended to be lower in IS98-3′NS2B/NS3/NS4A/NS4B-infected mice than for other tested viruses. Furthermore, at 12 days p.i. the mortality rate of mice infected with IS98-3′NS2B/NS3/NS4A/NS4B or WN-IT08 was similar (80%), and inferior to that of mice infected with WN-IS98 (100%), thus confirming the attenuated phenotype of this chimeric construct. In contrast, while IS98-NS5 showed efficient early replication compared to WN-IS98 and WN- IT08 *in vitro*, this result was not confirmed *in vivo*. IS98-3′NS4B/NS5-infected mice exhibited a viremia as high as that of mice infected with parental viruses, even if the mortality rate of IS98-3′NS4B/NS5-infected mice was closer to that of WN-IT08. In conclusion, IS98-3′NS2B/NS3/NS4A/NS4B and IS98-3′NS4B/NS5 displayed a degree of attenuation comparable to that of WN-IT08 and lower than that of the highly virulent WN-IS98, both confirming our previous findings suggesting a role for NS5 and 3′UTR in determining virulence in mice and serving to identify additional virulence determinants in the 3′NS2B/NS3/NS4A/NS4B region (Fiacre et al., 2020).

IS98-3′NS2B/NS3/NS4A/NS4B displayed an attenuated phenotype *in vitro* on *Vero* cells and *in vivo* in BALB/cByJ mice. We previously reported (Fiacre et al., 2023) that IS98-NS4A/NSAB/5′NS5 showed an attenuated phenotype in SPF chickens but not mice. Our new findings suggest an essential role for 3′NS2B and NS3 genomic regions as well in and in modulating replication efficacy in mammals, presumably by determining interactions between viral components (protein or RNA) and cellular components. The role of specific mutations differing between WN-IS98 and WN-IT08 in 3′NS2B and NS3, namely, NS3-P496L and NS3-E521D, in WNV virulence should be further studied. This chimeric construct exhibited other non-synonymous substitutions located in NS1 [NS1-N207I known as a molecular factor implicated in virulence attenuation in mammals (Whiteman et al., 2010), and probably generated during *SuperFi* polymerase amplification in the ISA process] and in the NS4/NS5 genomic regions (NS4A-A85I, NS4A-P100S, and NS5-S54P).

Comparison of WN-IS98 and WN-IT08 genomes revealed 22 non-synonymous mutations, including E-V159I, also found on IS98-NS1, and NS4B-E249D. Kobayashi et al. (2020), demonstrated that E-I159V increased virulence in a mouse model. Here, the attenuated phenotype of WN-IT08 in mammals could be due to the E-V159I or the NS4B-E249D mutations, as well as to other modifications discussed earlier. In a previous study investigating chimeras between WN-IS98 and WN-IT08 focusing on the 3′ end of the genome (NS4A, NS4B, NS5, and 3′UTR regions), we showed that the NS4B-249 residue could be implicated in slightly decreased virulence in mice (Fiacre et al., 2023). However, we showed in Fiacre et al. (2023), that the IS98-NS4A/NS4B/5′NS5 chimera containing NS4B-E249G was not markedly attenuated in mice. Here, IS98-NS4B/NS5 containing NS4A from WN-IS98 is attenuated in the mice model, suggesting a role for cumulative molecular changes in host virulence and a possible role for NS4A in the modulation of virulence in mammals.

In order to gain deeper insight into the viral determinants modulating mosquito-borne transmission, we performed experiments in insect models, including *in vitro* assays in the C6/36 mosquito cell line and competence studies in *Cx. pipiens* mosquitoes. Replication kinetics performed in C6/36 cells are indicative of an attenuated phenotype of IS98-3′UTR, as evidenced by lower viral RNA quantities and a lower production of infectious virions at 17 h post-infection, compared to the parental viruses and chimera IS98-3′NS4B/NS5. Of note, IS98-3′NS4B/NS5 and IS98-3′NS2B/NS3/NS4A/NS4B, which demonstrated reduced virulence in mice, did not show reduced replication in insect cells. We further assessed the vector competence of *Cx. pipiens* mosquitoes with WN-IS98, WN-IT08, IS98-3′UTR and IS98-3′NS4B/NS5. Vector competence is defined as the ability of an arthropod to transmit a pathogen after infection. This combines the intrinsic ability of the virus to successfully enter, replicate within the vector, disseminate, and finally replicate in the salivary glands before being released at a concentration that suffices for infection of a naïve vertebrate host. Even if IS98-3′UTR and IS98-3′NS4B/NS5 infected less *Cx. pipiens* than WN-IS98 and WN-IT08, they disseminated as efficiently as their parental strains. Moreover, the transmission rate of chimeric constructs was 50% lower than that of parental strains. Our results imply that the 3′UTR and the NS5 genomic regions of WN-IS98 are essential virulence factors implicated in the infection of *Cx. pipiens* and the release of the virus in the saliva. All together, these results suggest that *Cx. pipiens* is less competent for the two chimeric viruses than for either parental viruses. The altered phenotype may result from the reassociation of genomic regions originating from two different parental strains leading to suboptimal functional interactions at the molecular level during the virus cycle or from single point mutations introduced unintentionally during the ISA procedure, even though such non-synonymous mutations have been infrequently documented when complete genome sequences of parental and chimeric constructs have been compared.

We hypothesize that the replacement of the 3′UTR region of WN-IS98 by the corresponding region in WN-IT08 altered genome interactions involved in the first steps of WNV replication in mosquitoes. The 5′ and 3′UTR can form conserved stem-loop structures (Brinton et al., 1986; Brinton and Dispoto, 1988) like the 5′CS/3′CSI (Hahn et al., 1987) or the 5′UAR/3′UAR interactions (Hahn et al., 1987). As no differences were observed in these regions in our two parental genomes, we propose that nt 10517, nt 10520, nt 10523, nt 10689, nt 10721, nt 10775, nt 10830, nt 10832, nt 10852, which differ between WN-IS98 and WN-IT08, could be responsible for the altered vector-pathogen interactions and reduced replication in *Cx. pipiens*. Moreover, IonTorrent sequencing of the IS98-3′UTR chimera revealed two substitutions, one non-synonymous (NS4B-C120F) and the other synonymous (NS5-N867N, that could also play a role in *Cx. pipiens* replication.

As previously discussed in Fiacre et al. (2023), the NS5 protein is composed of many important conserved sequences known to be implicated in viral replication. However, no differences in these regions were observed between the sequences of WN-IS98 and WN-IT08. Other mutations found in WN-IT08 *vs.* WN-IS98 could be responsible for differences in replicative capacity in mammals and mosquitoes, such as NS5-H53Y, NS5-S54P, NS5-V258A, NS5-N280K, NS5-A732V and NS5-R422K. Moreover, IS98-3′NS4B/NS5 also contains the NS4B-E249G mutation identified in WN-IT08. The NS4B-249 residue is known to modulate WNV virulence in birds and mammals (Davis et al., 2004), but was also identified by Van Slyke et al. (2013) as a molecular marker implicated in enhanced transmission in *Cx. tarsalis*, in addition to prM-V156I and NS5-A804V (Davis et al., 2004). We demonstrated significantly enhanced transmission of IS98-3′NS4B/NS5 in comparison with parental viruses and IS98-3′UTR, which may question a role for NS4B-E249G in promoting the crossing of the midgut epithelium by WNV in *Cx. pipiens* mosquitoes.

Other non-synonymous mutations between WN-IS98 and IT08 have been previously shown to modify WNV replication in mosquitoes. As previously shown by Moudy et al. (2007), E protein modification at amino acid 159 (E-U159C, found in WN02) can be implicated in a shortening of the extrinsic incubation period. Our study did not reveal any difference in the replication capacity of WN-IS98 and WN-IT08 in C6/36 cells or in *Cx. pipiens*, suggesting that E-V159I cannot alone be responsible for modulating vector replication.

To conclude, our study improves knowledge about the molecular determinants that are responsible for the attenuated phenotype of WN-IT08 in mammalian hosts, namely, 3′NS2B/NS3/NS4A/NS4B and possibly NS3-P496L, NS3-E521D, NS4A-A85I and NS4A-P100S mutations. We cannot completely exclude the possibility that creation of the chimeras induced new mutations that possibly modulated mammalian virulence, such as the well-known molecular determinant NS1-N207A or the mutation NS5-S54P. As reported previously in Fiacre et al. (2023), NS5 mutations present in WN-IT08 can, through genomic interactions, participate in WNV attenuation. We also showed that no differences in vector competence were detected for WN-IS98 and WN-IT08, despite the fact that NS4B/NS5 and possibly the NS4B-E249G substitution improved dissemination in *Cx. pipiens*. Infection and transmission of viruses by *Culex* vectors seem to be influenced by interaction of NS4B/NS5 genes or proteins with the mosquito. Our results also suggest a role for the 3′UTR in vector infection/transmission, especially nt 10517, 10520, 10523, 10689, 10721, 10775, 10830, 10832 and 10852.

Finally, our study extends our understanding of genetic factors that may influence virulence of WN-IS98 and WN-IT08, two European WNV lineage 1 isolates differing in virulence for vertebrate hosts. This study has several limits. First, it was carried out on custom-generated chimeric viruses in order to modify and study the pathobiological properties of a considered virus. Secondly, we were constrained by the initial genetic differences between the WNV strains studied, with several mutations, non-synonymous or not, per fragment exchanged. Finally, the virulence factors of interest in vertebrate hosts differ between mammalian and avian species as described in Fiacre et al., 2020. An assessment of viral fitness and virulence need to be carried out on the considered species, modeling reservoir or clinically-susceptible hosts relevant for WNV epidemiology. Further studies are needed to understand the parallels and differences between infection dynamics in vectors and virulence in vertebrate hosts, and to identify vulnerable targets at every step of the WNV transmission cycle amenable to therapeutic and prophylactic interventions.

## Data availability statement

The original contributions presented in the study are included in the article/supplementary material, further inquiries can be directed to the corresponding authors.

## Ethics statement

The animal study was approved by the Joint Anses-UPEC-Alfort Veterinary School ethics committee under the permit number 2022-11-15-11. The study was conducted in accordance with the local legislation and institutional requirements.

## Author contributions

LF: Data curation, Formal analysis, Investigation, Methodology, Writing—original draft. AN: Methodology, Writing—review and editing. CM: Methodology, Writing—review and editing. MB: Validation, Writing—review and editing. MC: Methodology, Writing—review and editing. MD: Methodology, Writing—review and editing. TH: Methodology, Writing—review and editing. AE: Formal analysis, Writing—review and editing. NP: Writing—review and editing. DV: Methodology, Writing—review and editing. JR: Writing—review and editing. A-BF: Methodology, Writing—review and editing. MV: Writing—review and editing, Methodology. EA: Funding acquisition, Resources, Supervision, Writing—review and editing. SL: Conceptualization, Data curation, Investigation, Methodology, Project administration, Resources, Supervision, Validation, Writing—review and editing, Funding acquisition. GG: Conceptualization, Data curation, Investigation, Methodology, Project administration, Resources, Supervision, Validation, Writing—review and editing.
